# Supplementary material for: Association of ABC Efflux Transporter Genetic Variants and Adverse Drug Reactions and Survival in Patients with Non-Small Lung Cancer
Source: Genes (Basel). 2025 Apr 15;16(4):453. doi: 10.3390/genes16040453 (PMC12026804; doi:10.3390/genes16040453)
Supplement: Supplementary file 1 [file genes-16-00453-s001.zip › genes-3554063-supplementary.pdf]

Supplementary Materials

Supplementary Figures

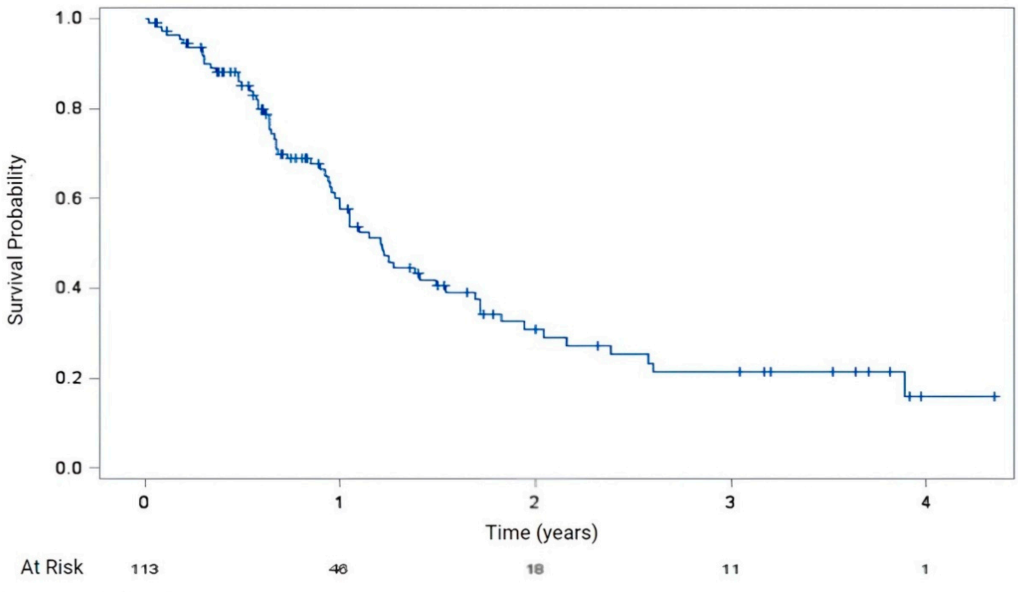

Figure S1: Overall survival as a function of time in years in non-small cell lung cancer patients treated with carboplatin and paclitaxel.

Supplementary Tables

Table S1. Clinical parameters used for evaluation of hematological, renal and hepatic adverse reactions in non-small cell lung cancer patients treated with carboplatin and paclitaxel.

**Clinical parameters**	**Total of patients (n)**	**Baseline (D0)**	**D20**	***p-value****
**Hematological parameters (mean ± SD)**				
Hemoglobin (g/L)	113	12.85 ± 1.71	12.10 ± 1.77	***<0.001***
Leukocytes ($x10^3/mm^3$)	113	9.23 ± 6.33	6.50 ± 3.07	***<0.001***
Neutrophils ($x10^3/mm^3$)	111	7.62 ± 8.70	6.34 ± 8.38	***<0.001***
Lymphocytes ($x10^3/mm^3$)	113	3.32 ± 3.98	3.59 ± 3.35	*0.292*
Platelets ($x10^3/mm^3$)	112	414.03 ± 536.98	273.49 ± 108.20	***0.0055***
**Renal parameters (mean ± SD)**				
Serum creatinine (mg/L)	112	0.80 ± 0.20	0.78 ± 0.18	*0.343*
Creatinine clearance**(mL/min)	111	89.11 ± 36.15	90.67 ± 26.89	*0.408*
Calcium (mg/dL)	99	9.22 ± 0.80	8.98 ± 0.72	***0.003***
Magnesium (mEq/L)	86	1.70 ± 0.18	1.59 ± 0.33	***0.017***
Potassium (mEq/L)	113	4.60 ± 0.63	4.55 ± 0.71	*0.110*
Sodium (mEq/L)	112	136.93 ± 3.23	136.56 ± 2.92	*0.172*
Inorganic phosphate (mg/dL)	88	3.83 ± 0.55	3.78 ± 0.84	*0.369*
Urea (mg/dL)	110	32.14 ± 13.16	29.33 ± 11.59	***0.006***
Uric acid (mg/dL)	105	4.62 ± 1.11	4.45 ± 1.31	*0.572*
**Hepatic parameters (mean ± SD)**				
Albumin (g/dL)	113	3.80 ± 0.50	3.76 ±0.48	*0.317*
TB (mg/dL)	108	0.40 ± 0.11	0.35 ± 0.11	***0.001***
GGT (U/L)	112	62.53 ± 76.48	70.09 ± 109.12	*0.397*
Total Proteins (g/dL)	82	6.84 ± 0.84	6.54 ± 0.70	***0.006***
ALP (U/L)	112	106.40 ± 52.06	107.16 ± 60.36	*0.824*
ALT (U/L)	112	19.31 ± 22.23	24.68 ± 43.77	*0.202*
AST (U/L)	112	17.45 ± 9.77	19.86 ± 15.79	*0.08*

N: Absolute number of patients; SD: standard deviation; D20: 20th day after the first cycle of carboplatin-paclitaxel chemotherapy; TB: total bilirubin; ALP: alkaline phosphatase; GGT: gamma-glutamyl transferase; ALT: alanine aminotransferase; AST: aspartate aminotransferase; *Friedman test. **Estimated by Cockroft-Gault equation.

Table S2. Frequency of *ABCB1* and *ABCC2* genotypes and alleles in non-small cell lung cancer patients treated with carboplatin and paclitaxel.

Gene	rs code	Nucleotide change	Classification	Allele or genotype	Allele frequency n (%)				
					**Study, n (%)**	**HWE**	**AbraOM (%)**	**GnomAD, all (%)**	***p-value***
ABCB1	rs1045642	c.3435A>G	*Missense*	AA	21 (19.1)	0.643	G= 58.5	G= 48.4	***<0,0001***
		p.Ile1215Met		AG	51 (46.4)				
				GG	38 (34.5)				
				A	42.3				
				G	57.7				
	rs1128503	c.1236C>T	Synonymous	TT	18 (16.4)	0.0821	C= 62.0	C= 55.9	***<0,0001***
		p.Gly412=		CT	42 (38.2)				
				CC	50 (45.5)				
				T	35.5				
				C	54.5				
	rs2032582	c.2677A>C/T	*Missense*	AA	11 (10.2)	0,146	C= 65.3<br>T = 7.5	C= 54.9<br>T= 2.6	***<0,0001***
		p.Ser893Ala		AC/CT/AT	49 (45.4)				
				CC	48 (44.4)				
				A	31.9				
				C	65.7				
				T	2.4				
ABCC2	rs717620	c.-24C>T	5 UTR	CC	77 (70.0)	0.970	T= 17.8	T= 18.6	*0,3411*
				CT	30 (27.3)				
				TT	3 (2.7)				
				C	85.0				
				T	15.0				

ABCB1: ATP Binding Cassette Subfamily B Member 1; *ABCC2*: ATP Binding Cassette Subfamily C Member 2; UTR: untranslated region; HWE: Hardy-Weinberg Equilibrium (>0.05); *AbraOM*: *Arquivo Brasileiro Online de Mutações* (https://abraom.ib.usp.br/index.php); *PharmGKB* (https://abraom.ib.usp.br/index.php), all populations (African, American, East Asian, European and South Asian).

Table S3. Univariate logistic regression associations between hematological adverse drug reactions, clinical and demographic data of non-small cell lung cancer patients treated with carboplatin and paclitaxel.

Variable	Hematological ADRs		*p-value*	OR	IC 95%
	No	Yes			
**Anemia**	N=74	N=39			
**Age (mean ± SD (N))**	62.64 ± 7.64	64.36 ± 6.58	*0.2341*	1.034	0.979;1.092
**Gender**					
Male	36 (60.0%)	24 (40.0%)	*0.1935*	1.689	0.767;3.721
Female	38 (71.7%)	15 (28.3%)			
**Ethnicity**					
White	60 (64.5%)	33 (35.5%)	*0.6403*	1.283	0.451;3.654
Non-white	14 (70.0%)	6 (30.0%)			
**Smoking status**					
Non-smoker	11 (64.7%)	6 (35.3%)	*0.9412*	0.960	0.326;2.828
Smoker	63 (65.6%)	33 (34.4%)			
**Alcoholism status**					
Abstainer	26 (68.4%)	12 (31.6%)	*0.6407*	1.219	0.531;2.797
Drinker	48 (64.0%)	27 (36.0%)			
**Comorbidities**					
Presence of comorbidities	44 (67.7%)	21 (32.3%)	*0.5663*	1.257	0.575;2.748
No comorbidities	30 (62.5%)	18 (37.5%)			
**Leukopenia**	N=93	N=20			
**Age (mean ± SD (N)**	63.28 ± 7.39	63.00 ± 7.09	*0.8761*	0.995	0.931;1.063
**Gender**					
Male	49 (81.7%)	11 (18.3%)	*0.8509*	1.098	0.416;2.896
Female	44 (83.0%)	9 (17.0%)			
**Ethnicity**					
White	76 (81.7%)	17 (18.3%)	*0.7279*	1.267	0.333;4.818
Non-white	17 (85.0%)	3 (15.0%)			
**Smoking status**					
Non-smoker	14 (82.4%)	3 (17.6%)	*0.9951*	1.004	0.260;3.884
Smoker	79 (82.3%)	17 (17.7%)			
**Alcoholism status**					
Abstainer	31 (81.6%)	7 (18.4%)	*0.8862*	0.929	0.337;2.562
Drinker	62 (82.7%)	13 (17.3%)			
**Comorbidities**					
Presence of comorbidities	54 (83.1%)	11 (16.9%)	*0.8015*	1.133	0.428;2.996
No comorbidities	39 (81.3%)	9 (18.8%)			
**Thrombocytopenia**	N=99	N=13			
**Age (mean ± SD (N))**	63.33 ± 7.2	62.38± 8.2	*0.6602*	0.983	0.909;1.063

Variable	Hematological ADRs		*p-value*	OR	IC 95%
	No	Yes			
**Gender**					
Male	54 (91.5%)	5 (8.5%)	*0.2807*	1.920	0.587;6.282
Female	45 (84.9%)	8 (15.1%)			
**Ethnicity**					
White	81 (87.1%)	12 (12.9%)	*0.3607*	2.666	0.326;21.831
Non-white	18 (94.7%)	1 (5.3%)			
**Smoking status**					
Non-smoker	14 (82.4%)	3 (17.6%)	*0.4041*	0.549	0.134;2.246
Smoker	85 (89.5%)	10 (10.5%)			
**Alcoholism status**					
Abstainer	32 (84.2%)	6 (15.8%)	*0.3268*	0.557	0.173;1.793
Drinker	67 (90.5%)	7 (9.5%)			
**Comorbidities**					
Presence of comorbidities	56 (87.5%)	8 (12.5%)	*0.7337*	0.814	0.249;2.665
No comorbidities	43 (89.6%)	5 (10.4%)			

Statistical analysis was performed using univariate Cox regressions. Statistically significant associations are in bold. N, absolute number of patients; OR, odds ratio; CI, confidence interval; SD, standard deviation; ADR, adverse drug reaction.

Table S4. Univariate logistic regression associations between hepatic adverse drug reactions, clinical, and demographic data of non-small cell lung cancer patients treated with carboplatin and paclitaxel.

**Variable**	**Hepatic ADRs**		***p-value***	**OR**	**IC 95%**
	**No**	**Yes**			
**Increased ALP**	N=96	N=16			
**Age (mean ± SD (N))**	62.95 ± 7.39	64.88 ± 7.01	*0.3307*	1.038	0.963;1.119
**Gender**					
Male	51 (86.4%)	8 (13.6%)	*0.8168*	1.133	0.393;3.267
Female	45 (84.9%)	8 (15.1%)			
**Ethnicity**					
White	78 (84.8%)	14 (15.2%)	*0.5488*	1.615	0.337;7.747
Non-white	18 (90.0%)	2 (10.0%)			
**Smoking status**					
Non-smoker	11 (64.7%)	6 (35.3%)	***0.0116***	4.637	1.409; 15.258
Smoker	85 (89.5%)	10 (10.5%)			
**Alcoholism status**					
Abstainer	33 (86.8%)	5 (13.2%)	*0.8070*	1.152	0.369;3.596
Drinker	63 (85.1%)	11 (14.9%)			
**Comorbidities**					
Presence of comorbidities	55 (85.9%)	9 (14.1%)	*0.9377*	1.043	0.359;3.034
No comorbidities	41 (85.4%)	7 (14.6%)			
**Hypoalbuminemia**	N=90	N=19			
**Age (mean ± SD (N))**	62.67 ± 7.16	66.79 ± 6.21	***0.0250***	1.091	1.011;1.178
**Gender**					
Male	46 (82.1%)	10 (17.9%)	*0.9041*	1.063	0.395;2.863
Female	44 (83.0%)	9 (17.0%)			
**Ethnicity**					
White	73 (82.0%)	16 (18.0%)	*0.7515*	1.242	0.325;4.749
Non-white	17 (85.0%)	3 (15.0%)			
**Smoking status**					
Non-smoker	14 (82.4%)	3 (17.6%)	*0.9795*	0.982	0.253; 3.822
Smoker	76 (82.6%)	16 (17.4%)			
**Alcoholism status**					
Abstainer	28 (73.7%)	10 (26.3%)	*0.0791*	0.406	0.149;1.110
Drinker	62 (87.3%)	9 (12.7%)			
**Comorbidities**					
Presence of comorbidities	50 (80.6%)	12 (19.4%)	*0.5442*	0.729	0.263;2.024
No comorbidities	40 (85.1%)	7 (14.9%)			

Statistical analysis was performed using univariate Cox regressions. Statistically significant associations are in bold. N, absolute number of patients; OR, odds ratio; CI, confidence interval; SD, standard deviation; ADR, adverse drug reaction.

Table S5. Univariate logistic regression associations between renal adverse drug reactions, clinical, and demographic data of non-small cell lung cancer patients treated with carboplatin and paclitaxel.

Variable	Renal ADRs		*p-value*	OR	IC 95%
	No	Yes			
**Hypocalcemia**	N=73	N=26			
**Age (mean ± SD (N)**	62.79 ± 6.64	67.04 ± 6.75	***0.0087***	1.102	1.025;1.185
**Gender**					
Male	36 (69.2%)	16 (30.8%)	*0.2860*	1.644	0.659;4.100
Female	37 (78.7%)	10 (21.3%)			
**Ethnicity**					
White	61 (73.5%)	22 (26.5%)	*0.9003*	0.924	0.270;3.169
Non-white	12 (75.0%)	4 (25.0%)			
**Smoking status**					
Non-smoker	13 (81.3%)	3 (18.8%)	*0.4593*	1.661	0.433; 6.371
Smoker	60 (72.3%)	23 (27.7%)			
**Alcoholism status**					
Abstainer	25 (71.4%)	10 (28.6%)	*0.6996*	0.833	0.330;2.104
Drinker	48 (75.0%)	16 (25.0%)			
**Comorbidities**					
Presence of comorbidities	39 (69.6%)	17 (30.4%)	*0.2931*	0.607	0.240;1.539
No comorbidities	34 (79.1%)	9 (20.9%)			
**Increased serum creatinine**	N=83	N=28			
**Age (mean ± SD (N)**	63.45 ± 7.73	62.64 ± 6.27	*0.6171*	0.985	0.930;1.044
**Gender**					
Male	47 (79.7%)	12 (20.3%)	*0.2093*	1.741	0.733;4.135
Female	36 (69.2%)	16 (30.8%)			
**Ethnicity**					
White	72 (78.3%)	20 (21.7%)	*0.0688*	2.618	0.928;7.383
Non-white	11 (57.9%)	8 (42.1%)			
**Smoking status**					
Non-smoker	12 (70.6%)	5 (29.4%)	*0.6663*	0.777	0.248; 2.441
Smoker	71 (75.5%)	23 (24.5%)			
**Alcoholism status**					
Abstainer	26 (68.4%)	12 (31.6%)	*0.2683*	0.608	0.252;1.467
Drinker	57 (78.1%)	16 (21.9%)			
**Comorbidities**					
Presence of comorbidities	47 (74.6%)	16 (25.4%)	*0.9620*	0.979	0.412;2.326
No comorbidities	36 (75.0%)	12 (25.0%)			
**Hypomagnesemia**	N=69	N=14			

Variable	Renal ADRs		*p-value*	OR	IC 95%
	No	Yes			
**Age (mean ± SD (N)**	63.04 ± 7.80	63.64 ± 6.33	*0.7851*	1.011	0.936;1.092
**Gender**					
Male	31 (77.5%)	9 (22.5%)	*0.1931*	2.206	0.670;7.265
Female	38 (88.4%)	5 (11.6%)			
**Ethnicity**					
White	59 (83.1%)	12 (16.9%)	*0.9840*	1.017	0.197;5.243
Non-white	10 (83.3%)	2 (16.7%)			
**Smoking status**					
Non-smoker	10 (76.9%)	3 (23.1%)	*0.5179*	0.621	0.147; 2.628
Smoker	59 (84.3%)	11 (15.7%)			
**Alcoholism status**					
Abstainer	25 (86.2%)	4 (13.8%)	*0.5850*	1.420	0.403;5.003
Drinker	44 (81.5%)	10 (18.5%)			
**Comorbidities**					
Presence of comorbidities	40 (85.1%)	7 (14.9%)	*0.5841*	1.379	0.436;4.363
No comorbidities	29 (80.6%)	7 (19.4%)			
**Hyponatremia**	N=89	N=24			
**Age (mean ± SD (N)**	63.17 ± 7.67	63.46 ± 5.93	*0.8625*	1.006	0.945;1.070
**Gender**					
Male	47 (78.3%)	13 (21.7%)	*0.9058*	1.056	0.427;2.609
Female	42 (79.2%)	11 (20.8%)			
**Ethnicity**					
White	71 (76.3%)	22 (23.7%)	*0.1910*	2.788	0.600; 12.969
Non-white	18 (90.0%)	2 (10.0%)			
**Smoking status**					
Non-smoker	11 (64.7%)	6 (35.3%)	*0.1319*	0.423	0.138; 1.295
Smoker	78 (81.3%)	18 (18.8%)			
**Alcoholism status**					
Abstainer	26 (68.4%)	12 (31.6%)	*0.0598*	0.413	0.164; 1.037
Drinker	63 (84.0%)	12 (16.0%)			
**Comorbidities**					
Presence of comorbidities	52 (80.0%)	13 (20.0%)	*0.7080*	1.189	0.480;2.945
No comorbidities	37 (77.1%)	11 (22.9%)			

Statistical analysis was performed using univariate Cox regressions. Statistically significant associations are in bold. N, absolute number of patients; OR, odds ratio; CI, confidence interval; SD, standard deviation; ADR, adverse drug reaction.

Table S6. Univariate logistic regression associations between gastrointestinal adverse drug reactions, clinical, and demographic data of non-small cell lung cancer patients treated with carboplatin and paclitaxel.

Variable	Gastrointestinal ADRs		*p-value*	OR	IC 95%
	**No**	**Yes**			
**Nausea**	N=83	N=30			
**Age (mean ± SD (N)**	63.14 ± 7.31	63.47 ± 7.42	*0.8354*	1.006	0.950;1.066
**Gender**					
Male	49 (81.7%)	11 (18.3%)	***0.0381***	2.489	1.051;5.894
Female	34 (64.2%)	19 (35.8%)			
**Ethnicity**					
White	69 (74.2%)	24 (25.8%)	*0.7003*	1.232	0.426;3.568
Non-white	14 (70.0%)	6 (30.0%)			
**Smoking status**					
Non-smoker	15 (88.2%)	2 (11.8%)	*0.1512*	3.088	0.662;14.397
Smoker	68 (70.8%)	28 (29.2%)			
**Alcoholism status**					
Abstainer	27 (71.1%)	11 (28.9%)	*0.6813*	0.833	0.348;1.994
Drinker	56 (74.7%)	19 (25.3%)			
**Comorbidities**					
Presence of comorbidities	50 (76.9%)	15 (23.1%)	*0.3322*	1.515	0.654;3.509
No comorbidities	33 (68.8%)	15 (31.3%)			
**Vomiting**	N=97	N=16			
**Age (mean ± SD (N)**	62.75 ± 7.36	66.13 ± 6.43	*0.0895*	1.071	0.989;1.159
**Gender**					
Male	54 (90,0%)	6 (10,0%)	*0.1835*	2.093	0.705;6.216
Female	43 (81.1%)	10 (18.9%)			
**Ethnicity**					
White	81 (87.1%)	12 (12.9%)	*0.4128*	1.688	0.482;5.903
Non-white	16 (80.0%)	4 (20.0%)			
**Smoking status**					
Non-smoker	17 (100,0%)	0 (0.0%)	*0.1241*		
Smoker	80 (83.3%)	16 (16,7%)			
**Alcoholism status**					
Abstainer	32 (84.2%)	6 (15.8%)	*0.7238*	0.821	0.274;2.458
Drinker	65 (86.7%)	10 (13.3%)			
**Comorbidities**					
Presence of comorbidities	56 (86.2%)	9 (13.8%)	*0.9115*	1.062	0.366;3.087
No comorbidities	41 (85.4%)	7 (14.6%)			

Variable	Gastrointestinal ADRs		*p-value*	OR	IC 95%
	No	Yes			
**Diarrhea**	N=96	N=17			
**Age (mean ± SD (N)**	62.78 ± 7.21	65.76 ± 7.59	*0.1227*	1.062	0.984;1.146
**Gender**					
Male	49 (81.7%)	11 (18.3%)	*0.3021*	1.759	0.602;5.138
Female	47 (88.7%)	6 (11.3%)			
**Ethnicity**					
White	78 (83.9%)	15 (16.1%)	*0.4912*	1.731	0.363;8.252
Non-white	18 (90.0%)	2 (10.0%)			
**Smoking status**					
Non-smoker	17 (100.0%)	0 (0.0%)	*0.0705*		
Smoker	79 (82.3%)	17 (17.7%)			
**Alcoholism status**					
Abstainer	34 (89.5%)	4 (10.5%)	*0.3437*	1.782	0.539;589
Drinker	62 (82.7%)	13 (17.3%)			
**Comorbidities**					
Presence of comorbidities	53 (81.5%)	12 (18.5%)	*0.2428*	0.514	0.168; 1.571
No comorbidities	43 (89.6%)	5 (10.4%)			

Statistical analysis was performed using univariate Cox regressions. Statistically significant associations are in bold. N, absolute number of patients; OR, odds ratio; CI, confidence interval; SD, standard deviation; ADR, adverse drug reaction.

Table S7. Univariate logistic regression analysis associating hepatic adverse drug reactions with *ABCB1* and *ABCC2* variants in non-small cell lung cancer patients treated with carboplatin and paclitaxel.

	**Hepatic ADRs**				
	**Grade 0** (*n*, %)	**Grade 1-4** (*n*, %)	***p-value***	**OR**	**95% CI**
**Increased ALP**	N=95	N=15			
***ABCB1* rs1045642 (c.3435A>G)**					
**Dominant model**					
AA (ref)	18 (90.0)	2 (10.0)	*0.5913*	1.539	0.319; 7.436
AG+GG	76 (85.4)	13 (14.6)			
**Recessive model**					
GG (ref)	35 (92.1)	3 (7.9)	*0.2037*	2.373	0.626; 8.994
AG+AA	59 (83.1)	12 (16.9)			
***ABCB1* rs1128503 (c.1236C>T)**					
**Dominant model**					
CC (ref)	19 (90.5)	2 (9.5)	*0.5339*	1.647	0.342; 7.927
CT+TT	75 (85.2)	13 (14.8)			
**Recessive model**					
TT (ref)	45 (91.8)	4 (8.2)	*0.1347*	2.525	0.750; 8.502
CT+CC	49 (81.7)	11 (18.3)			
***ABCB1* rs2032582 (c.2677A>C/T)**					
CC (ref)	43 (91.5)	4 (8.5)	*0.1554*	2.413	0.716; 8.137
Non-CC (TT+AA+TA+CA+CT)	49 (81.7)	11 (18.3)			
AA (ref)	10 (90.9)	1 (9.1)	*0.6229*	1.707	0.202; 14.400
Non-AA (TT+CC+AT+CA+CT)	82 (85.4)	14 (14.6)			
***ABCC2* rs717620 (c.-24C>T)**					
**Dominant model**					
CC (ref)	69 (89.5)	8 (10.5)	*0.1440*	2.288	0.754;6,947
CT+TT	26 (78.8)	7 (21.2)			
**Recessive model**					
TT (ref)	1 (33.3)	2 (66.7)	***0.0347***	14.308	1.211;169.086
CT+CC	93 (87.7)	13 (12.3)			
**Hypoalbuminemia**	**N=90**	**N=19**			
***ABCB1* rs1045642 (c.3435A>G)**					
**Dominant model**					
AA (ref)	15 (78.9)	4 (21.1)	*0.6952*	1.280	0.372; 4.403
AG+GG	72 (82.8)	15 (17.2)			
**Recessive model**					
GG (ref)	31 (83.8)	6 (16.2)	*0.7373*	1.199	0.415; 3.470
AG+AA	56 (81.2)	13 (18.8)			

	**Hepatic ADRs**				
	**Grade 0** (*n*, %)	**Grade 1-4** (*n*, %)	***p-value***	**OR**	**95% CI**
***ABCB1* rs1128503 (c.1236C>T)**					
**Dominant model**					
CC (ref)	19 (95.0)	1 (5.0)	*0.1274*	5.029	0.630;40.133
CT+TT	68 (79.1)	18 (20.9)			
**Recessive model**					
TT (ref)	40 (83.3)	8 (16.7)	*0.7589*	1.170	0.429; 3.192
CT+CC	47 (81.0)	11 (19.0)			
***ABCB1* rs2032582 (c.2677A>C/T)**					
CC (ref)	38 (82.6)	8 (17.4)	*0.8366*	1.112	0.406; 3.040
Non-CC (TT+AA+AT+CA+CT)	47 (81.0)	11 (19.0)			
AA (ref)	10 (100.0)	0 (0.0)	*0.2018*	-	
Non-AA (TT+CC+AT+CA+CT)	75 (79.8)	19 (20.2)			
***ABCC2* rs717620 (c.-24C>T)**					
**Dominant model**					
CC (ref)	60 (80.0)	15 (20.0)	*0.3899*	1.687	0.512; 5.562
CT+TT	27 (87.1)	4 (12.9)			
**Recessive model**					
TT (ref)	2 (66.7)	1 (33.3)	*0.4926*	2.361	0.203;27.464
CT+CC	85 (82.5)	18 (17.5)			

Statistical analysis was performed by univariate logistic regressions. Statistically significant associations are in bold. ADR, adverse drug reaction; N, absolute number of patients; OR, odds ratio; 95% CI, 95% confidence interval; *ABCB1*, ATP Binding Cassette Subfamily B Member; *ABCC2*: ATP Binding Cassette Subfamily C Member 2.

Table S8: Univariate logistic regression analysis associating gastrointestinal adverse drug reactions with *ABCB1 and ABCC2* variants in non-small cell lung cancer patients treated with carboplatin and paclitaxel.

	**Gastrointestinal ADRs**				
	**Grade 0** (*n*, %)	**Grade 1-4** (*n*, %)	***p-value***	**OR**	**95% CI**
**Nausea**	N=83	N=30			
***ABCB1* rs1045642 (c.3435A>G)**					
**Dominant model**					
AA (ref)	15 (75.0)	5 (25.0)	*0.8784*	1.091	0.358;3.326
AG+GG	66 (73.3)	24 (26.7)			
**Recessive model**					
GG (ref)	33 (84.6)	6 (15.4)	*0.0580*	2.635	0.968;7.177
AA+AG	48 (67.6)	23 (32.4)			
***ABCB1* rs1128503 (c.1236C>T)**					
**Dominant model**					
CC (ref)	18 (85.7)	3 (14.3)	*0.1732*	2.476	0.672;9.130
CT+TT	63 (70.8)	26 (29.2)			
**Recessive model**					
TT (ref)	43 (86.0)	7 (14.0)	***0.0093***	3.556	1.367;9.250
CC+CT	38 (63.3)	22 (36.7)			
***ABCB1* rs2032582 (c.2677A>C/T)**					
CC (ref)	39 (81.3)	9 (18.8)	*0.0929*	2.167	0.879;5.339
Non-CC (TT+AT+AA+CA+CT)	40 (66.7)	20 (33.3)			
AA (ref)	11 (100.0)	0 (0.0)	***0.0341***	-	
Non-AA (TT+AT+CC+CA+CT)	68 (70.1)	29 (29.9)			
***ABCC2* rs717620 (c.-24C>T)**					
**Dominant model**					
CC (ref)	54 (70.1)	23 (29.9)	*0.2023*	1.917	0.698; 5.264
CT+TT	27 (81.8)	6 (18.2)			
**Recessive model**					
TT (ref)	2 (66.7)	1 (33.3)	*1.0000*	1.411	0.123; 16.168
CT+CC	79 (73.8)	28 (26.2)			
**Vomiting**	N=96	N=14			
***ABCB1* rs1045642 (c.3435A>G)**					
**Dominant model**					
AA (ref)	17 (85.0)	3 (15.0)	*0.7364*	0.789	0.199;3.136
AG+GG	79 (87.8)	11 (12.2)			
**Recessive model**					
GG (ref)	37 (94.9)	2 (5.1)	*0.0943*	3.763	0.797;17.770
AG+AA	59 (83.1)	12 (16.9)			

	Gastrointestinal ADRs				
	**Grade 0** (*n*, %)	**Grade 1-4** (*n*, %)	***p-value***	**OR**	**95% CI**
***ABCB1* rs1128503 (c.1236C>T)**					
**Dominant model**					
CC (ref)	20 (95.2)	1 (4.8)	*0.2494*	3.421	0.422; 27.735
CT+TT	76 (85.4)	13 (14.6)			
**Recessive model**					
TT (ref)	49 (98.0)	1 (2.0)	***0.0137***	13.553	1.705; 107.723
CC+CT	47 (78.3)	13 (21.7)			
***ABCB1* rs2032582 (c.2677A>C/T)**					
CC (ref)	44 (91.7)	4 (8.3)	*0.2083*	2.200	0.644; 7.514
Non-CC (TT+AT+AA+CA+CT)	50 (83.3)	10 (16.7)			
AA (ref)	11 (100.0)	0 (0.0)	*0.3525*	-	-
Non-AA (TT+AT+CC+CA+CT)	83 (85.6)	14 (14.4)			
***ABCC2* rs717620 (c.-24C>T)**					
**Dominant model**					
CC (ref)	67 (87.0)	10 (13.0)	*0.9007*	1.082	0.314; 3.734
CT+TT	29 (87.9)	4 (12.1)			
**Recessive model**					
TT (ref)	3 (100.0)	0 (0.0)	*1.0000*	-	-
CT+CC	93 (86.9)	14 (13.1)			
**Diarrhea**	(N=96)	(N=17)			
***ABCB1* rs1045642 (c.3435A>G)**					
**Dominant Model**					
AA (ref)	18 (90.0)	2 (10.0)	*0.5275*	1.658	0.346; 7.954
AG+GG	76 (84.4)	14 (15.6)			
**Recessive Model**					
GG (ref)	34 (87.2)	5 (12.8)	*0.7041*	1.247	0.400; 3.889
AG+AA	60 (84.5)	11 (15.5)			
***ABCB1* rs1128503 (c.1236C>T)**					
**Dominant model**					
CC (ref)	21 (100.0)	0 (0.0)	***0.0383***	-	-
CT+TT	73 (82.0)	16 (18.0)			
**Recessive model**					
TT (ref)	44 (88.0)	6 (12.0)	*0.4912*	1.467	0.493; 4.363
CC+CT	50 (83.3)	10 (16.7)			
***ABCB1* rs2032582 (c.2677A>C/T)**					
CC (ref)	41 (85.4)	7 (14.6)	*0.9518*	1.034	0.355; 3.013
Non-CC (TT+AT+AA+CA+CT)	51 (85.0)	9 (15.0)			

	Gastrointestinal ADRs				
	**Grade 0** (*n*, %)	**Grade 1-4** (*n*, %)	***p-value***	**OR**	**95% CI**
AA (ref)	11 (100.0)	0 (0.0)	*0.3634*	-	-
Non-AA (TT+|AT+CC+CA+CT)	81 (83.5)	16 (16.5)			
***ABCC2* rs717620 (c.-24C>T)**					
**Dominant model**					
CC (ref)	65 (84.4)	12 (15.6)	*0.6378*	1.338	0.398;4.503
CT+TT	29 (87.9)	4 (12.1)			
**Recessive model**					
TT (ref)	3 (100.0)	0 (0.0)	*1.0000*	-	
CT+CC	91 (85.0)	16 (15.0)			

Statistical analysis was performed by univariate logistic regressions. Statistically significant associations are in bold. ADR, adverse drug reaction; N, absolute number of patients; OR, odds ratio; 95% CI, 95% confidence interval; *ABCB1*, ATP Binding Cassette Subfamily B Member; *ABCC2*: ATP Binding Cassette Subfamily C Member 2.

Table S9: Univariate logistic regression analysis associating hematological adverse drug reactions with *ABCB1* and *ABCC2* variants in non-small cell lung cancer patients treated with carboplatin and paclitaxel.

	**Hematological ADRs**				
	**Grade 0** (*n*, %)	**Grade 1-4** (*n*, %)	***p-value***	**OR**	**95% CI**
**Anemia**	**N=74**	**N=39**			
***ABCB1* rs1045642 (c.3435A>G)**					
**Dominant model**					
AA (ref)	13 (65.0)	7 (35.0)	*0.9622*	1.025	0.371; 2.832
AG+GG	59 (65.6)	31 (34.4)			
**Recessive model**					
GG (ref)	25 (64.1)	14 (35.9)	*0.8251*	0.912	0.402; 2.067
AG+AA	47 (66.2)	24 (33.8)			
***ABCB1* rs1128503 (c.1236C>T)**					
**Dominant model**					
CC (ref)	13 (61.9)	8 (38.1)	*0.7039*	0.826	0.309; 2.211
CT+TT	59 (66.3)	30 (33.7)			
**Recessive model**					
TT (ref)	36 (72.0)	14 (28.0)	*0.1894*	1.714	0.766; 3.834
CT+CC	36 (60.0)	24 (40.0)			
***ABCB1* rs2032582 (c.2677A>C/T)**					
CC (ref)	34 (70.8)	14 (29.2)	*0.4121*	1.406	0.623; 3.174
Non-CC (TT+AA+TA+CA+CT)	38 (63.3)	22 (36.7)			
AA (ref)	8 (72.7)	3 (27.3)	*0.6539*	1.375	0.342; 5.530
Non-AA (TT+CC+AT+CA+CT)	64 (66.0)	33 (34.0)			
***ABCC2* rs717620 (c.-24C>T)**					
**Dominant model**					
CC (ref)	51 (66.2)	26 (33.8)	*0.7930*	0.892	0.381; 2.092
CT+TT	21 (63.6)	12 (36.4)			
**Recessive model**					
TT (ref)	2 (66.7)	1 (33.3)	*0.9646*	1.057	0.093;12.040
CT+CC	70 (65.4)	37 (34.6)			
**Leukopenia**	**N=93**	**N=20**			
***ABCB1* rs1045642 (c.3435A>G)**					
**Dominant model**					
AA (ref)	17 (85.0)	3 (15.0)	*0.7666*	1.225	0.320;4.684
AG+GG	74 (82.2)	16 (17.8)			
**Recessive model**					
GG (ref)	33 (84.6)	6 (15.4)	*0.6982*	1.233	0.428;3.549
AG+AA	58 (81.7)	13 (18.3)			
***ABCB1* rs1128503 (c.1236C>T)**					

	Hematological ADRs		*p-value*	OR	95% CI
	**Grade 0** (*n*, %)	**Grade 1-4** (*n*, %)			
**Dominant model**					
CC (ref)	19 (90.5)	2 (9.5)	*0.3070*	2.243	0.476; 10.567
CT+TT	72 (80.9)	17 (19.1)			
**Recessive model**					
TT (ref)	44 (88.0)	6 (12.0)	*0.1872*	2.028	0.709; 5.803
CT+CC	47 (78.3)	13 (21.7)			
ABCB1 **rs2032582 (c.2677A>C/T)**					
CC (ref)	41 (85.4)	7 (14.6)	*0.4642*	1.464	0.527;4.065
Non-CC (TT+AA+TA+CA+CT)	48 (80.0)	12 (20.0)			
AA (ref)	11 (100.0)	0 (0.0)	*0.2065*	-	
Non-AA (TT+CC+AT+CA+CT)	78 (80.4)	19 (19.6)			
ABCC2 **rs717620 (c.-24C>T)**					
**Dominant model**					
CC (ref)	64 (83.1)	13 (16.9)	*0.8689*	1.094	0.376;3.179
CT+TT	27 (81.8)	6 (18.2)			
**Recessive model**					
TT (ref)	3 (100.0)	0 (0.0)	*1.0000*	-	
CT+CC	88 (82.2)	19 (17.8)			
**Thrombocytopenia**	**N=99**	**N=13**			
ABCB1 **rs1045642 (c.3435A>G)**					
**Dominant model**					
AA (ref)	19 (95.0)	1 (5.0)	*0.3113*	2.960	0.362;24.179
AG+GG	77 (86.5)	12 (13.5)			
**Recessive model**					
GG (ref)	32 (82.1)	7 (17.9)	*0.1558*	2.333	0.724; 7.519
AG+AA	64 (91.4)	6 (8.6)			
ABCB1 **rs1128503 (c.1236C>T)**					
**Dominant model**					
CC (ref)	18 (85.7)	3 (14.3)	*0.7110*	1.300	0.324; 5.211
CT+TT	78 (88.6%)	10 (11.4)			
**Recessive model**					
TT (ref)	43 (86.0)	7 (14.0)	*0.5402*	1.438	0.450; 4.597
CT+CC	53 (89.8)	6 (10.2)			
ABCB1 **rs2032582 (c.2677A>C/T)**					
CC (ref)	43 (89.6)	5 (10.4)	*0.6216*	1.349	0.411; 4.429
Non-CC (TT+AA+TA+CA+CT)	51 (86.4)	8 (13.6)			

	Hematological ADRs		*p-value*	OR	95% CI
	**Grade 0** (*n*, %)	**Grade 1-4** (*n*, %)			
AA (ref)	10 (90.9)	1 (9.1)	*0.7446*	1.428	0.168;12.163
Non-AA (TT+CC+AT+CA+CT)	84 (87.5)	12 (12.5)			
***ABCC2* rs717620 (c.-24C>T)**					
**Dominant model**					
CC (ref)	66 (86.8%)	10 (13.2%)	*0.5494*	1.515	0.389; 5.906
CT+TT	30 (90.9%)	3 (9.1%)			
**Recessive model**					
TT (ref)	3 (100.0%)	0 (0.0%)	*1.0000*	-	
CT+CC	93 (87.7%)	13 (12.3%)			

Statistical analysis was performed by univariate logistic regressions. Statistically significant associations are in bold. ADR, adverse drug reaction; N, absolute number of patients; OR, odds ratio; 95% CI, 95% confidence interval; *ABCB1*, ATP Binding Cassette Subfamily B Member; *ABCC2*: ATP Binding Cassette Subfamily C Member 2.

Table S10: Univariate logistic regression analysis associating renal adverse drug reactions with *ABCB1* and *ABCC2* variants in non-small cell lung cancer patients treated with carboplatin and paclitaxel.

	**Renal ADRs**				
	**Grade 0** (*n*, %)	**Grade 1-4** (*n*, %)	***p-value***	**OR**	**95% CI**
**Hypocalcemia**	**N=73**	**N=26**			
***ABCB1* rs1045642 (c.3435A>G)**					
**Dominant model**					
AA (ref)	12 (63.2)	7 (36.8)	*0.2235*	1.944	0.667; 5.673
AG+GG	60 (76.9)	18 (23.1)			
**Recessive model**					
GG (ref)	23 (71.9)	9 (28.1)	*0.7104*	1.198	0.461; 3.115
AG+AA	49 (75.4)	16 (24.6)			
***ABCB1* rs1128503 (c.1236C>T)**					
**Dominant model**					
CC (ref)	14 (77.8)	4 (22.2)	*0.7032*	1.267	0.375; 4.284
CT+TT	58 (73.4)	21 (26.6)			
**Recessive model**					
TT (ref)	31 (73.8)	11 (26.2)	*0.9345*	1.039	0.415; 2.600
CT+CC	41 (74.5)	14 (25.5)			
***ABCB1* rs2032582 (c.2677A>C/T)**					
**Dominant model**					
CC (ref)	31 (77.5)	9 (22.5)	*0.4724*	1.413	0.550; 3.628
Non-CC (TT+AA+TA+CA+CT)	39 (70.9)	16 (29.1)			
AA (ref)	8 (80.0)	2 (20.0)	*0.6336*	1.483	0.293; 7.508
Non-AA (TT+CC+AT+CA+CT)	62 (72.9)	23 (27.1)			
***ABCC2* rs717620 (c.-24C>T)**					
**Dominant model**					
CC (ref)	47 (69.1)	21 (30.9)	*0.0865*	2.792	0.863; 9.035
CT+TT	25 (86.2)	4 (13.8)			
**Recessive model**					
TT (ref)	3 (100.0)	0 (0.0)	*0.5666*	-	
CT+CC	69 (73.4)	25 (26.6)			
**Increased serum creatinine**	N=83	N=28			
***ABCB1* rs1045642 (c.3435A>G)**					
**Dominant model**					
AA (ref)	16 (80.0)	4 (20.0)	*0.5051*	1.500	0.455; 4.938
AG+GG	64 (72.7)	24 (27.3)			
**Recessive model**					

	Renal ADRs		*p-value*	OR	95% CI
	**Grade 0** (*n*, %)	**Grade 1-4** (*n*, %)			
GG (ref)	28 (75.7)	9 (24.3)	*0.7840*	1.137	0.455; 2.843
AG+AA	52 (73.2)	19 (26.8)			
***ABCB1* rs1128503 (c.1236C>T)**					
**Dominant model**					
CC (ref)	17 (81.0)	4 (19.0)	*0.4260*	1.619	0.494; 5.302
CT+TT	63 (72.4)	24 (27.6)			
**Recessive model**					
TT (ref)	33 (68.8)	15 (31.3)	*0.2608*	0.609	0.256; 1.446
CT+CC	47 (78.3)	13 (21.7)			
***ABCB1* rs2032582 (c.2677A>C/T)**					
CC (ref)	31 (67.4)	15 (32.6)	*0.2078*	1.749	0.733; 4.176
Non-CC (TT+AA+TA+CA+CT)	47 (78.3)	13 (21.7)			
AA (ref)	10 (90.9)	1 (9.1)	*0.1988*	3.971	0.485;32.536
Non-AA (TT+CC+AT+CA+CT)	68 (71.6)	27 (28.4)			
***ABCC2* rs717620 (c.-24C>T)**					
**Dominant model**					
CC (ref)	56 (74.7)	19 (25.3)	*0.8323*	1.105	0.438;2.790
CT+TT	24 (72.7)	9 (27.3)			
**Recessive model**					
TT (ref)	3 (100.0)	0 (0.0)	*0.5666*	-	
CT+CC	77 (73.3)	28 (26.7)			
**Hypomagnesemia**					
***ABCB1* rs1045642 (c.3435A>G)**					
**Dominant model**					
AA (ref)	11 (78.6)	3 (21.4)	*0.6529*	1.389	0.332; 5.808
AG+GG	56 (83.6)	11 (16.4)			
**Recessive model**					
GG (ref)	24 (77.4)	7 (22.6)	*0.3247*	1.792	0.561; 5.719
AG+AA	43 (86.0)	7 (14.0)			
***ABCB1* rs1128503 (c.1236C>T)**					
**Dominant model**					
CC (ref)	15 (93.8)	1 (6.3)	*0.2203*	3.750	0.453; 31.040
CT+TT	52 (80.0)	13 (20.0)			
**Recessive model**					
TT (ref)	30 (85.7)	5 (14.3)	*0.5351*	1.459	0.442; 4.819
CT+CC	37 (80.4)	9 (19.6)			
***ABCB1* rs2032582 (c.2677A>C/T)**					
CC (ref)	27 (79.4)	7 (20.6)	*0.5630*	1.407	0.442;4.481
Non-CC (TT+AA+TA+CA+CT)	38 (84.4)	7 (15.6)			

	Renal ADRs				
	**Grade 0** (*n*, %)	**Grade 1-4** (*n*, %)	***p-value***	**OR**	**95% CI**
AA (ref)	8 (100.0)	0 (0.0)	*0.3383*	-	
Non-AA (TT+CC+AT+CA+CT)	57 (80.3)	14 (19.7)			
ABCC2 **rs717620 (c.-24C>T)**					
**Dominant model**					
CC (ref)	48 (84.2)	9 (15.8)	*0.5845*	1.404	0.416;4.733
CT+TT	19 (79.2)	5 (20.8)			
**Recessive model**					
TT (ref)	2 (100.0)	0 (0.0)	*1.0000*	-	
CT+CC	65 (82.3)	14 (17.7)			
**Hyponatremia**	**N=89**	**N=24**			
ABCB1 **rs1045642 (c.3435A>G)**					
**Dominant model**					
AA (ref)	18 (90.0)	2 (10.0)	*0.1732*	2.911	0.625;13.551
AG+GG	68 (75.6)	22 (24.4)			
**Recessive model**					
GG (ref)	29 (74.4)	10 (25.6)	*0.4729*	1.404	0.556; 3.546
AG+AA	57 (80.3)	14 (19.7)			
ABCB1 **rs1128503 (c.1236C>T)**					
**Dominant model**					
CC (ref)	17 (81.0)	4 (19.0)	*0.7329*	1.232	0.372; 4.080
CT+TT	69 (77.5)	20 (22.5)			
**Recessive model**					
TT (ref)	40 (80.0)	10 (20.0)	*0.6737*	1.217	0.487; 3.041
CT+CC	46 (76.7)	14 (23.3)			
ABCB1 **rs2032582 (c.2677A>C/T)**					
CC (ref)	38 (79.2)	10 (20.8)	*0.7563*	1.157	0.462; 2.897
Non-CC (TT+AA+TA+CA+CT)	6 (76.7)	14 (23.3)			
AA (ref)	11 (100.0)	0 (0.0)	*0.1181*	-	
Non-AA (TT+CC+AT+CA+CT)	73 (75.3)	24 (24.7)			
ABCC2 **rs717620 (c.-24C>T)**					
**Dominant model**					
CC (ref)	59 (76.6)	18 (23.4)	*0.5466*	1.373	0.490; 3.845
CT+TT	27 (81.8)	6 (18.2)			
**Recessive model**					
TT (ref)	3 (100.0)	0 (0.0)	*1.0000*	-	
CT+CC	83 (77.6)	24 (22.4)			

Statistical analysis was performed by univariate logistic regressions. Statistically significant associations are in bold. ADR, adverse drug reaction; N, absolute number of patients; OR, odds ratio; 95% CI, 95% confidence interval; *ABCB1*, ATP Binding Cassette Subfamily B Member; *ABCC2*: ATP Binding Cassette Subfamily C Member 2.

Table S11: Survival analysis associated with demographic and clinical data of non-small cell lung cancer patients treated with carboplatin and paclitaxel.

**Variable**	**Death**		***p-value***	**HR**	**CI 95%**
	**No**	**Yes**			
**Age (mean ± SD (N))**	62.78 ± 7.21 (N=96)	65.76 ± 7.59 (N=17)	*0.1705*	1.027	0.989;1.068
**Gender, n (%)**					
Male	21 (35.0)	39 (65.0)	*0.0558*	1.632	0.988;2.697
Female	27 (50.9)	26 (49.1)			
**Ethnicity, n (%)**					
White	40 (43.0)	53 (57.0)	*0.4918*	1.247	0.664;2.341
Non-white	8 (40.0)	12 (60.0)			
**Smoking status, n (%)**					
Non-smoker	11 (64.7)	6 (35.3)	***0.0006***	5.048	2.004;12.711
Smoker	37 (38.5)	59 (61.5)			
**Alcoholism status, n (%)**					
Abstainer	17 (44.7)	21 (55.3)	*0.1809*	1.433	0.846;2.426
Drinker	31 (41.3)	44 (58.7)			
**Comorbidities, n (%)**					
Presence of comorbidities	24 (36.9)	41 (63.1)	*0.8013*	0.937	0.566; 1.553
No comorbidities	24 (50.0)	24 (50.0)			

Statistical analysis was performed using univariate Cox regressions. CI: confidence interval; HR: hazard ratio; SD: standard deviation.

Table S12. Univariate Cox regressions between adverse drug reactions and death of non-small cell lung cancer patients treated with carboplatin and paclitaxel.

Variable	Death		*p-value*	HR	IC 95%
	No (N=48)	Yes (N=65)			
**Anemia, n (%)**					
Grade 0	33 (44.6)	41 (55.4)	*0.8985*	0.967	0.582;1.608
Grade 1	15 (38.5)	24 (61.5)			
**Leukopenia, n (%)**					
Grade 0	41 (44.1)	52 (55.9)	*0.9688*	0.988	0.537;1.816
Grade 1	7 (35.0)	13 (65.0)			
**Thrombocytopenia, n (%)**					
Grade 0	44 (44.4)	55 (55.6)	*0.6038*	1.206	0.594;2.448
Grade 1	4 (30.8)	9 (69.2)			
**Hypoalbuminemia, n (%)**					
Grade 0	42 (46.7)	48 (53.3)	*0.9730*	1.010	0.554;1.844
Grade 1	5 (26.3)	14 (73.7)			
**ALP increase, n (%)**					
Grade 0	45 (46.9)	51 (53.1)	*0.4667*	1.246	0.689;2.256
Grade 1	2 (12.5)	14 (87.5)			
**Reduced creatinine clearance, n (%)**					
Grade 0	37 (44.6)	46 (55.4)	*0.8999*	0.966	0.559;1.669
Grade 1	10 (35.7)	18 (64.3)			
**Hypomagnesemia, n (%)**					
Grade 0	29 (42.0)	40 (58.0)	*0.7253*	1.133	0.563;2.280
Grade 1	4 (28.6)	10 (71.4)			
**Hypocalcemia, n (%)**					
Grade 0	38 (52.1)	35 (47.9)	***0.022***	2.317	1.353;3.967
Grade 1	4 (15.4)	22 (84.6)			
**Hyponatremia, n (%)**					
Grade 0	39 (43.8)	50 (56.2)	*0.2383*	0.701	0.389;1.265
Grade 1	9 (37.5)	15 (62.5)			
**Nausea, n (%)**					
Grade 0	39 (47.0)	44 (53.0)	*0.0766*	1.604	0.951;2.704
Grade 1	9 (30.0)	21 (70.0)			
**Vomiting, n (%)**					
Grade 0	43 (44.3)	54 (55.7)	***0.0013***	3.047	1.548;5.997
Grade 1	5 (31.3)	11 (68.8)			
**Diarrhea, n (%)**					
Grade 0	44 (45.8)	52 (54.2)	***0.0006***	2.974	1.590; 5.562
Grade 1	4 (23.5)	13 (76.5)			

Statistical analysis was performed using univariate Cox regressions.; CI: confidence interval; HR: hazard ratio; SD: standard deviation.

Table S13: Survival analysis associated with *ABCB1* and *ABCC2* variants in non-small cell lung cancer patients treated with carboplatin and paclitaxel.

Genotypes	Death No N (%)	Death Yes N (%)	*p-value*	HR	95% CI
***ABCB1* rs1045642 (A>G)**					
**Dominant Model**					
AA (ref)	6 (30.0)	14 (70.0)	*0.1251*	1.604	0.877; 2.936
AG+GG	40 (44.4)	50 (55.6)			
**Recessive Model**					
GG (ref)	17 (43.6)	22 (56.4)	*0.0763*	1.599	0.952; 2.686
AG+AA	29 (40.8)	42 (59.2)			
***ABCB1* rs1128503 (C>T)**					
**Dominant Model**					
CC (ref)	7 (33.3)	14 (66.7)	*0.6699*	1.138	0.627;2.066
CT+TT	39 (43.8)	50 (56.2)			
**Recessive Model**					
TT (ref)	26 (52.0)	24 (48.0)	***0.0361***	1.725	1.036; 2.871
CT+CC	20 (33.3)	40 (66.7)			
***ABCB1* rs2032582 (C>A/T)**					
CC (ref)	25 (52.1)	23 (47.9)			
Non-CC (TT+AA+AT+CA+CT)	20 (33.3)	40 (66.7)	***0.0265***	1.795	0.836; 3.784
AA (ref)	3 (27.3)	8 (72.7)	*0.1350*	1.779	0.836;3.784
Non-AA (TT+AT+CC+CA+CT)	42 (43.3)	55 (56.7)			
***ABCC2* rs717620 (C>T)**					
**Dominant Model**					
CC (ref)	32 (41.6)	45 (58.4)	*0.4936*	1.206	0.705; 2.063
CT+TT	14 (42.4)	19 (57.6)			
**Recessive Model**					
TT (ref)	2 (66.7)	1 (33.3)	*0.2959*	2.876	0.397;20.839
CT+CC	44 (41.1)	63 (58.9)			

HR: hazard ratio; CI: confidence interval; *ABCB1*: ATP Binding Cassette Subfamily B Member 1; *ABCC2*: ATP Binding Cassette.
